# Large scale chemical patent mining with UIMA and UNICORE

**DOI:** 10.1186/1758-2946-4-S1-P19

**Published:** 2012-05-01

**Authors:** Alexander Klenner, Sandra Bergmann, Marc Zimmermann, Mathilde Romberg

**Affiliations:** 1 Fraunhofer-Institute for Algorithms and Scientific Computing (SCAI), Sankt Augustin, 53754, Germany; 2 Forschungszentrum Juelich GmbH, Juelich, 52425, Germany

## 

Finding information about annotated chemical reactions for drugs and small compounds is a crucial step for pharmaceutical industries. This data often is presented in form of unstructured documents (especially patents) and manual extraction of this information is a time- and cost inefficient effort.

In our project UIMA-HPC [1], we describe the combined usage of Unstructured Information Managment Architecture (UIMA) and Uniform Interface to Computing Recources (UNICORE) for large-scale chemical patent mining. Our approach will incorporate existing software such as chemoCR for image processing (image-to-structure) and OCR for text reconstruction. All components are wrapped inside the UIMA framework pipeline. Using the UIMA framework ensures compatibility between different components of the pipeline and makes it possible to connect arbitrary annotation modules into this system. Scale-out for large document collections is achieved by the UNICORE framework on High Performance Clusters, which enables parallelization of all UIMA nodes. The aim is a fully annotated pdf collection where all biomedical entities (compound names, reaction schemes, etc.) are connected by references and thus can be easily browsed and searched by the user. Planned schematic workflow is shown in Figure 1.

**Figure 1:**
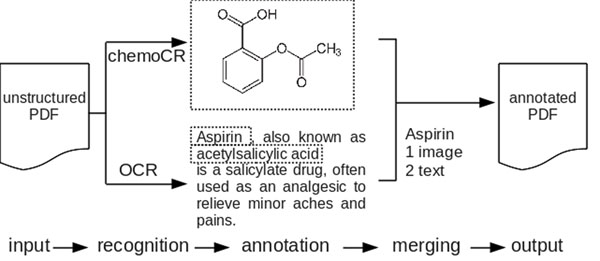
Planned workflow of our UIMA framework. 'Recognition' and 'annotation' are CPU intensive parts that are parallelized on demand using the UNICORE framework. 'Merging' checks for cross-annotations (entity in text and image). Finally, an annotated PDF is presented as output.

## Funding

BMBF grant 01IH1101.
